# The association between motor capacity and mobility performance: frailty as a moderator

**DOI:** 10.1186/s11556-019-0223-4

**Published:** 2019-10-10

**Authors:** Carl-Philipp Jansen, Nima Toosizadeh, M. Jane Mohler, Bijan Najafi, Christopher Wendel, Michael Schwenk

**Affiliations:** 1 Network Aging Research, Heidelberg University, Bergheimer Straße 20, 69115 Heidelberg, Germany; 2 Department of Biomedical Engineering & Medicine, University of Arizona, Tucson, USA; 3 Interdisciplinary Consortium on Advanced Motion Performance (iCAMP), Baylor College of Medicine, Houston, TX USA

**Keywords:** Frailty, Motor capacity, Mobility performance, Moderation analysis, Wearable sensors, ICF

## Abstract

**Background:**

In older adults, the linkage between laboratory-assessed ‘motor capacity’ and ‘mobility performance’ during daily routine is controversial. Understanding factors moderating this relationship could help developing more valid assessment as well as intervention approaches. We investigated whether the association between capacity and performance becomes evident with transition into frailty, that is, whether frailty status moderates their association.

**Methods:**

We conducted a cross-sectional analysis of the observational (blinded for review) study in a community-dwelling cohort in (blinded for review). Participants were *N* = 112 older adults aged 65 years or older who were categorized as non-frail (*n* = 40), pre-frail (*n* = 53) or frail (*n* = 19) based on the Fried frailty index.

Motor capacity was quantified as normal (NWS) and fast walking speed (FWS). Mobility performance was quantified as 1) cumulated physical activity (PA) time and 2) everyday walking performance (average steps per walking bout; maximal number of steps in one walking bout), measured by a motion sensor over a 48 h period. Hierarchical linear regression analyses were performed to evaluate moderation effects.

**Results:**

Unlike in non-frail persons, the relationship between motor capacity and mobility performance was evident in pre-frail and frail persons, confirming our hypothesis. A moderating effect of frailty status was found for 1) the relationship between both NWS and FWS and maximal number of steps in one bout and 2) NWS and the average steps per bout. No moderation was found for the association between NWS and FWS with cumulated PA.

**Conclusion:**

In pre-frail and frail persons, motor capacity is associated with everyday walking performance, indicating that functional capacity seems to better represent mobility performance in this impaired population. The limited relationship found in non-frail persons suggests that other factors account for their mobility performance. Our findings may help to inform tailored assessment approaches and interventions taking into consideration a person’s frailty status.

## Introduction

‘Motor capacity’ refers to an individual’s motor function assessed in a standardized laboratory environment whereas ‘mobility performance’ depicts enacted mobility in real-life situations [1]. The International Classification of Functioning, Disability, and Health (ICF) differentiates between these two measures: what a person can do (capacity) and does do (performance). Understanding the association and complementarity of motor capacity and mobility performance could help to understand gait, balance, and mobility disabilities in older adults. If a relationship exists, one could use motor capacity measures as a surrogate marker of mobility performance. Likewise, a causal relationship would imply that improving a person’s motor capacity would result in increased mobility performance. However, research has shown that the association between motor capacity and motor performance is not straightforward, and several studies have shown that in-lab measured gait differs from real-life measured gait in community-dwelling older adults [2–6]. One reason may be that younger or healthy older adults need a lower relative effort compared to impaired older individuals who perform near their maximal capacity to execute daily motor tasks [7]. In support of this idea, laboratory studies have shown this for muscle function [8] and walking [4, 9], which led us to the assumption that the association between motor capacity and mobility performance might become evident with increasing impairment in older persons. This assumption has not been tested, yet. It also remains unclear whether this has implications for everyday life.

Frailty incorporates both muscle function and walking and is a widely used, accepted cumulative measure of age-related, gradual multisystem impairment [10, 11]. We therefore use frailty status as a distinguishing criterion, categorizing older persons into different stages of impairment, which allows to further explore the association of motor capacity in mobility performance in different subsamples. Specifically, we investigated whether the association between motor capacity and mobility performance is moderated by frailty status in older adults. We hypothesized that the association becomes more evident with transition into frailty status.

## Methods

### Study design and participants

This study is a secondary analysis of baseline data collected in the (blinded for review), an observational descriptive study conducted in (blinded for review) among community-dwelling older adults aged 65 years or older (elaborate sample and recruitment description elsewhere [12–14]). Eligible subjects gave informed consent approved by the Institutional Review Board of the (blinded for review).

### Frailty assessment

Participants were categorized as non-frail, pre-frail, and frail based on the frailty phenotype [15], which includes five criteria. Slowness was determined based on participants’ time to perform the 4.57 m walk test. Weakness was assessed using a hand dynamometer. Low energy expenditure was assessed using the Minnesota Leisure Time Activity Questionnaire [16]. Exhaustion was determined based on two items of the Center for Epidemiological Studies Depression Scale [17]. Unintentional weight loss was defined as self-reported loss of > 4.54 kg over the past year. Norm-based scoring was done using a computerized scoring algorithm in accordance with Fried et al. [15]. For each criterion, participants were scored either 0 or 1; a sum score of 0 was defined as non-frail; 1–2 as pre-frail; ≥3 as frail.

### Sensor-based monitoring of mobility performance

Over 48 h daily PA was monitored using an unobtrusive, shirt-embedded sensor (PAMSys™, BioSensics LLC, Watertown, MA, USA) based on algorithms validated in geriatric populations (described elsewhere [18–20]). The sensor identifies body postures, postural transitions, and walking; a walking bout was defined as a minimum of three consecutive steps. The following parameters representing enacted mobility performance were used for analyses: cumulated proportion of time spent in everyday PA (percentage of total time either walking or standing) as a general measure of PA; average number of steps per walking bout and maximal number of steps in one walking bout as more specific measures related to everyday functioning and independence.

### Sensor-based assessment of motor capacity

Walking speed has predictive value for falls, fractures, hospitalization, and mortality [21, 22]. It is usually measured as ‘normal walking speed’ and ‘maximum speed’ to acknowledge the necessity to walk at different speeds and modify gait speed [23]. Both parameters reflect a different aspect of capacity, as NWS is what one would expect being performed in a real-life environment and fast walking speed as the maximal capacity of a person in a true sense. Hence, motor capacity was operationalized as normal (NWS) and fast (FWS) walking speed. It was assessed using commercially available, wearable sensors (LEGSys™; BioSensics, Cambridge, Mass., USA). The system consists of five inertial sensors attached to shank, thighs, and lower back; gait parameters were derived using validated algorithms [24, 25]. To assess NWS, participants walked a distance of 4.57 m at self-selected speed in their home, if possible without walking aids. The same procedure was applied to assess FWS with a distance of up to 10 m, giving the instructions to walk as quickly as possible, but safely.

### Covariates

Self-reported age, sex (only NWS models), body mass index (BMI), and number of self-reported comorbidities were included as covariates.

### Statistical analyses

A moderation analysis was performed to examine for what condition (frailty status) the association between motor capacity and mobility performance exists, and in what magnitude (detailed information on moderation analysis can be found elsewhere [26]). In other words, it was analysed how frailty changes the main effect of motor capacity on mobility performance. Hierarchical linear regression was used with each mobility performance outcome as the dependent variable. Frailty status was dummy-coded into two variables for NWS with reference to the non-frail group, i.e., a pre-fail and a frail dummy. For FWS analyses, frailty status was dichotomised into non-frail and pre-frail/frail because only three frail persons were able to perform the FWS test.

In Step 1 of the regression analyses, potential confounders (see covariates) and main effects of motor capacity outcomes were entered into the model. We reduced the number of confounding variables in the FWS analyses in order to not overload the statistical model. In Step 2, frailty dummies (NWS models) or dichotomous frailty status (FWS models) were entered. Interaction terms (motor capacity variables×frailty status) were entered in Step 3; if these are significant predictors, a moderation is present Statistical significance was set to *p* = .05 throughout all analyses. In case of non-normally distributed residuals, variables were squared or log10-transformed depending on their skewness. In case of significant interactions, models were probed using the PROCESS macro [26], and 5000 bootstrapped samples were drawn to calculate 95% confidence intervals. Statistical analyses were performed using SPSS (IBM SPSS Statistics, Version 24.0; Armonk, NY, USA).

## Results

The final sample for NWS consisted of *n* = 112 subjects of which 40 were categorized as non-frail, 53 as pre-frail and 19 as frail. On average, 78.8% were women (range across groups: 73.0–89.5), aged 78.8 years (range 74.7–82.8) with a BMI of 27.7 (range 25.8–29.9); had 3.5 chronic conditions (range 2.3–4.6); spent a cumulated 20.7% in PA (range 16.4–25.0); had an average of 923 maximal steps in one walking bout (range 285–1668); averaged 34 steps per walking bout (range 27–39); had a NWS of 0.96 m/s (range 0.64–1.18); and a FWS of 1.24 m/s (range 1.07–1.47). Baseline characteristics of the three groups are shown in Table 1.

**Table 1:** Participant characteristics

	Non-frail	Pre-frail	Frail	*P* ^†^
N	40	53	19	
BMI, mean ± SD, kg/m^2^	25.8 ± 4.4	28.4 ± 6.9	29.9 ± 6.0	.026
Age, mean ± SD, years	74.7 ± 6.6	80.5 ± 8.7	82.8 ± 8.8	<.001
Sex, % female	85.0	73.6	89.5	.214^§^
Number of comorbidities, mean ± SD	2.3 ± 1.7	4.0 ± 2.2	4.6 ± 1.6	<.001
Cumulated PA, mean ± SD, %	25.0 ± 7.1	18.9 ± 6.0	16.4 ± 7.3	<.001
Max. steps in one bout, mean ± SD	1668 ± 1724	591 ± 556	285 ± 357	<.001
Aver. steps per bout, mean ± SD	39 ± 24	33 ± 15	27 ± 12	.025
Normal walking speed, mean ± SD m/s	1.18 ± 0.15	0.92 ± 0.22	0.64 ± 0.25	<.001
Fast walking speed, mean ± SD, m/s	1.47 ± 0.22^a^	1.13 ± 0.27^b^	1.07 ± 0.12^c^	<.001

*BMI* Body mass index, *max* Maximal, *PA* Physical activity, *SD* Standard deviation

^†^: one-way ANOVA; ^§^: Pearson Chi^2^

^a^: *n* = 29; ^b^: *n* = 33; ^c^: *n* = 3

### Regression of normal walking speed on mobility performance outcomes

**Fig. 1:**
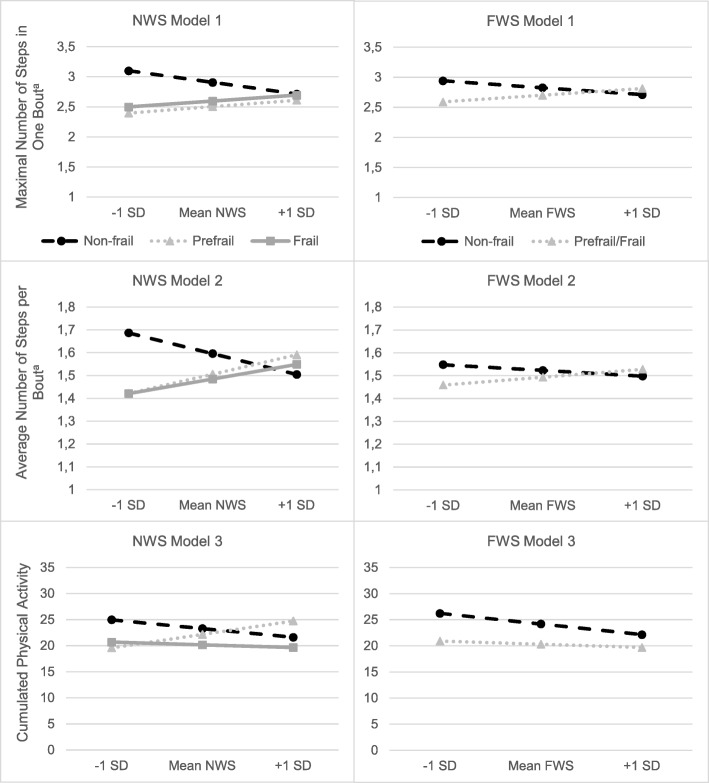
Regression lines depicting the relationship between mobility performance and motor capacity (normal and fast walking speed) at specified values within each frailty group. Legend: Model 1: Maximal number of steps in one bout; Model 2: average number of steps per bout; (Model 3, in percent) cumulated physical activity; ^a^: log-transformed data; FWS: fast walking speed; NWS: normal walking speed; SD: standard deviation*.* In non-frail, average number of steps per bout and maximal number of steps in one bout decline from low to high NWS and FWS, i.e., faster walkers have a smaller amount of maximal number of steps in one bout and average number of steps per bout; in pre-frail and frail subjects maximal number of steps in one bout and average number of steps per bout incline from low to high NWS and FWS. Regarding the cumulated physical activity, an incline is only shown for one subgroup (prefrail) at NWS, but not at FWS

Regression results are shown in Table 2 and depicted Fig. 1.

**Table 2:** Multiple stepwise regression of NWS on mobility performance outcomes

	∆*R*^2^	B	SE_B_	β	t	*p*
Maximal Number of Steps in One Bout^a^						
Step 1	.285					<.001
(constant)		3.84	.42		9.20	<.001
NWS		.38	.15	.28	2.58	.011
Step 2	.031					.101
(constant)		3.81	.41		9.20	<.001
NWS		.15	.18	.11	.85	.400
dummy 1 pre-frail		−.13	.09	−.17	−1.46	.148
dummy 2 frail		−.29	.13	−.28	−2.17	.033
Step 3	.048					.024
(constant)		3.98	.41		9.75	<.001
NWS		−.70	.35	−.51	−1.98	.050
dummy 1 pre-frail		−.29	.10	−.39	−2.82	.006
dummy 2 frail		−.38	.16	−.38	−2.37	.020
dummy 1 x NWS		1.08	.46	.47	2.35	.021
dummy 2 x NWS		1.05	.40	.42	2.64	.010
total *R*^2^ = .364 (*N* = 112; *p* = < .001)						
Average Number of Steps per Bout^a^						
Step 1	.137					.007
(constant)		1.90	.25		7.67	<.001
NWS		.17	.09	.23	1.90	.056
Step 2	.002					.869
(constant)		1.90	.25		7.58	<.001
NWS		.14	.11	.19	1.27	.206
dummy 1 pre-frail		−.01	.05	−.02	−.15	.883
dummy 2 frail		−.04	.08	−.07	−.48	.633
Step 3	.051					.044
(constant)		1.99	.25		8.02	<.001
NWS		−.33	.21	−.45	−1.54	.126
dummy 1 pre-frail		−.10	.06	−.25	−1.59	.115
dummy 2 frail		−.08	.10	−.14	−.79	.430
dummy 1 x NWS		.63	.28	.51	2.27	.026
dummy 2 x NWS		.56	.24	.42	2.32	.022
total *R*^2^ = .190 (*N* = 112; *p* = .008)						
Cumulated PA						
Step 1	.427					<.001
(constant)		62.08	7.31		8.50	<.001
NWS		2.48	2.57	.09	.96	.338
Step 2	.008					.461
(constant)		61.58	7.36		8.37	<.001
NWS		.20	3.19	.01	.06	.950
dummy 1 pre-frail		−1.61	1.54	−.11	−1.05	.298
dummy 2 frail		−2.82	2.34	−.14	− 1.20	.231
Step 3	.021					.139
(constant)		62.71	7.37		8.51	<.001
NWS		−6.07	6.35	−.23	−.96	.341
dummy 1 pre-frail		−3.05	1.87	−.21	−1.63	.106
dummy 2 frail		−.85	2.91	−.04	−.29	.772
dummy 1 x NWS		15.41	8.31	.34	1.85	.067
dummy 2 x NWS		4.21	7.19	.09	.59	.560
total *R*^2^ = .457 (*N* = 112; *p* < .001)						

Step 1 to 3 controlled for age, sex, number of comorbidities, and bmi

*NWS* Normal walking speed

^a^: log-transformed

#### Model 1: NWS and maximal number of steps in one walking bout

Step 1 (*R*^2^ = .285; *p* < .001) was significant, showing a direct effect of NWS. In Step 2 (∆*R*^2^ = .031; *p* = .101) the direct effect disappeared in presence of frailty status dummy variables. In Step 3, both interaction terms significantly contributed to the model (∆*R*^2^ = .048; *p* = .024; pre-frail×NWS: B = 1.08, *p* = .021; frail×NWS: B = 1.05, *p* = .010), i.e., a significant moderation was shown. Effects remained significant after bootstrapping, indicating robust estimates for the interaction of NWS with pre-frail (95% CI 0.26–1.84) and frail (95% CI 0.17–2.00). Interestingly, the overall direct effect of NWS (Step 1) is positive (B = .38), but when including the interaction terms in Step 3 it becomes clear that this is due to the frail and pre-frail group; in non-frail the direction is negative (B = -.70) (see also Fig. 1).

#### Model 2: NWS and average number of steps per walking bout

Step 1 (*R*^2^ = .137; *p* = .007) indicating the crude effect of NWS was significant. In Steps 2 and 3, NWS and both dummies did not contribute significantly to the model (∆*R*^2^ = .002; *p* = .869). However, in Step 3 the interaction was significant for both terms (pre-frail×NWS: B = .63, *p* = .026; frail×NWS: B = .56, *p* = .022; ∆*R*^2^ = .051; *p* = .044) which means a significant moderation by frailty status. Estimates were robust after bootstrapping: pre-frail (95% CI: 0.04–1.25) and frail (95% CI: 0.07–1.12). Regarding the direction of the effects the same as in model 1 was observed.

#### Model 3: NWS and cumulated time spent in PA

Factors included in Step 1 explained a large proportion of the variance in cumulated PA (*R*^2^ = .427; *p* < .001). In Step 2 and 3 none of the included variables added significantly to the overall model; no moderation effect was observed.

### Regression of fast walking speed on mobility performance outcomes

This sub-sample consisted of 29 non-frail and 36 pre-frail/frail subjects. Regression results for FWS are shown in Table 3 and depicted in Fig. 1.

**Table 3:** Multiple stepwise regression of FWS on mobility performance outcomes

	∆*R*^2^	B	SE_B_	β	t	*p*
Maximal Number of Steps in One Bout^a^						
Step 1	.258					.001
(constant)		3.81	.64		6.00	<.001
FWS		.13	.17	.11	.76	.449
Step 2	.004					.575
(constant)		3.85	.64		5.99	<.001
FWS		−.09	.19	.07	.47	.639
frailty status dichot.		−.06	.11	−.08	−.56	.575
Step 3	.055					.033
(constant)		4.42	.68		6.53	<.001
FWS		−1.14	.60	−.92	−1.92	.060
frailty status dichot.		−.40	.19	−.54	−2.13	.037
frailty stat. x FWS		.76	.35	.85	2.18	.033
total *R*^2^ = .317 (*N* = 66; *p* = .001)						
Average Number of Steps per Bout^a^						
Step 1	.130					.070
(constant)		1.98	.37		5.34	<.001
FWS		.50	.10	.08	.49	.625
Step 2	.001					.834
(constant)		1.99	.38		5.29	<.001
FWS		.04	.11	.06	.36	.718
frailty status dichot.		−.01	.06	−.03	−.21	.834
Step 3	.013					.350
(constant)		2.14	.41		5.23	<.001
FWS		−.28	.36	−.42	−.78	.437
frailty status dichot.		−.10	.11	−.26	−.90	.373
frailty stat. x FWS		.20	.21	.41	.94	.350
total *R*^2^ = .144 (*N* = 66; *p* = .149)						
Cumulated PA						
Step 1	.379					<.001
(constant)		70.39	11.43		6.16	<.001
FWS		−1.40	3.14	−.06	−.45	.657
Step 2	.032					.074
(constant)		72.78	11.30		6.44	<.001
FWS		−3.83	3.36	.16	−1.14	.259
frailty status dichot.		−3.49	1.92	−.24	−1.82	.074
Step 3	.006					.455
(constant)		76.38	12.31		6.21	<.001
FWS		−11.56	10.83	−.47	− 1.07	.290
frailty status dichot.		−5.63	3.44	−.39	−1.64	.107
frailty stat. x FWS		4.76	6.33	.27	.75	.455

total *R*^2^ = .417 (*N* = 66; *p* < .001)

Step 1 to 3 controlled for age, number of comorbidities, and bmi

*FWS* Fast walking speed

^a^: log-transformed

#### Model 1: FWS and maximal number of steps in one walking bout

There was no overall direct effect of FWS in Step 1. In Step 2 and Step 3, FWS and frailty status did not contribute significantly to the model as well, but the interaction in Step 3 was significant (B = .76, *p* = .033), that is, a moderation by frailty status is present. This effect also remained after bootstrapping (95% CI: .06–1.45).

#### Model 2: FWS and average number of steps per walking bout

This association was not moderated by frailty status (∆*R*^2^ = .013; *p* = .350). The overall model showed neither direct effects of FWS nor an interaction, indicating that this overall weak association (*R*^2^ = .144; *p* = .149) persists independently of frailty status.

#### Model 3: FWS and cumulated time spent in PA

This association was not moderated by frailty status (∆*R*^2^ = .006; *p* = .455). Direct effects of FWS or frailty status were not observed; the overall model (*R*^2^ = .417; *p* < .001) still was significant, indicating that the included confounding variables explained a rather large proportion of the variance.

## Discussion

To our knowledge, this is the first study that has investigated whether the association between motor capacity and mobility performance is moderated by frailty status. We have confirmed our hypothesis that frailty status indeed acts as a moderator of this relationship. Against the background of inconclusive results of previous studies showing that these factors are weakly associated or not associated in different samples [2, 3] we have enhanced this line of research with the important finding that they in fact are associated when using one of the most accepted categorization—frailty status according to Fried et al.—as a distinguishing criterion. More precisely, motor capacity is only associated with gait-related mobility performance in daily life if a certain degree of physiological impairment is given, in our case pre-frailty and frailty. No significant associations were found in the non-frail group in the final models (Step 3), with trends in the counterintuitive direction that higher capacity is associated with lower performance. One explanation could be that there is a ‘performance threshold’ where higher capacity does not yield any performance enhancement; or that persons with higher motor capacity have larger variability in their mobility performance, which would lead to lower correlations between both measures.

Unlike in the other two mobility performance outcomes, the moderation effect was not evident in CumPA, that is, the association between motor capacity and mobility performance was not related to frailty status. Simply put, the moderation effect is present with regard to *how* someone enacts her/his mobility, but not with regard to *how much* she/he is physically active during daily life in general. This is backed by findings from van Lummel et al. [27] who found that physical activity and physical performance described two different domains of physical function. Others have shown that slower walking speed is associated with less physical activity according to activity monitoring [28]. This inconsistency may be explained with the fact that factors affecting mobility are complex [29, 30], which may be the case especially when a more behaviour-oriented aspect of mobility such as physical activity is investigated.

Our findings hold important practical implications and suggest laboratory-based gait assessment better represents walking-related everyday performance in pre-frail and frail older persons. For example, being able to walk longer distances and to perform several walking bouts of a somewhat longer distance may have a strong impact on the degree of autonomy and self-determination in everyday life: Participation in recreational activities within the neighbourhood or upholding social contacts within the community would be doable without assistance. In clinical practice, motor capacity assessments (e.g., timed up-and-go, gait speed) are used to draw conclusions on subjects’ mobility performance and functionality in real life, reflecting their performance beyond the time of the assessment. This is critical as clinical decisions or subsequent therapy prescriptions are often based on such laboratory-based test results.

Based on our findings we can only speculate about a causal relationship between both factors, that is, the question whether actual mobility performance may be improved via increasing motor capacity (walking speed) in frail persons warrants further research. A confirmed causal relationship would allow tailored interventions depending on frailty status.

Important strengths of our study are the objective, sensor-based assessment of mobility performance and motor capacity using validated systems as well as the innovative character of our investigation. Still, our results should be interpreted with respect to potential limitations. Since walking speed is a frailty criterion, there is a theoretical contamination in the data. To explore a potential circular reasoning a modified version of the frailty phenotype was calculated as previously done by Blodgett et al., [31]. When analyses were rerun with this modified frailty phenotype, no moderation effects were found. However, sample sizes were severely changed by this modification: for NWS models, 45% (FWS: 31%) more subjects were categorized as non-frail than with the ‘normal’ frailty phenotype, that is, many of those who were by definition pre-frail were now labelled as non-frail. Moreover, analysis of multicollinearity between NWS/FWS and frailty dummies showed that neither variance inflation factor nor tolerance of the models were above generally accepted limits [32]. We suggest this issue be investigated in future research, e.g., by measuring motor capacity using multi-dimensional mobility tasks. Another limitation is that in our sample, only 19 subjects were categorized as frail. Hence, statistical power to detect a moderating effect may have been limited in this sample. For FWS, we have solved this matter (frail: *n* = 3) by dichotomizing frailty status into non-frail and pre-frail/frail, which appears to be justified given how close the coefficients were for pre-frail and frail in the NWS models 1 and 2. Another limitation is that our sample was predominantly women, which is why we have controlled for sex in the models of NWS, but not FWS in order not to ‘overload’ the models due to the smaller sample size. Regarding frailty, there is no overall consensus on an operative definition [33]; possibly a broader frailty concept or a different measure could have altered the results. We also want to highlight that the effect sizes of the interactions are rather small in most models. In some models, the direct effect explains far more variance than the interaction whereas in others the proportion of variance explained by the interaction terms is rather large (e.g., Table 2, 2nd model, more than a quarter of the overall variance explanation), and no other direct effects are observed. This is no unexpected result because one can expect the lab-measured capacity to explain a fair amount of variance of real life performance. However, the significant interaction shows that the grouping variable (frailty status) significantly impacts on the amount of variance explained, that is, we could confirm our hypothesis that the issue of frailty–or maybe other impairments as well–moderating the relationship between motor capacity and mobility performance is worth considering in future research. A next step could be to examine a moderation effect of the associations between the exact same parameters when measured in the laboratory and during real life, as performed by Hillel et al. [5]. Qualitative outcomes such as gait variability, symmetry, regularity, and other outcomes that represent gait quality may hold promising potential for future research as well.

## Conclusions

In conclusion, the association between motor capacity and mobility performance is only present in pre-frail and frail subjects, indicating that functional capacity measures seem to better represent mobility performance in these two groups than in non-frail persons. The limited relationship found in in non-frail persons suggests that there must be other factors accounting for mobility performance. In addition, our findings may help to inform the development of tailored assessment approaches and intervention paradigms taking into consideration a person’s frailty status. As a final conclusion, this can be seen as a first step towards the establishment of a framework of the association between motor capacity and mobility performance.

## Data Availability

The data that support the findings of this study are available from JM, but restrictions apply to the availability of these data, which were used under license for the current study, and so are not publicly available. Data are however available from the authors upon reasonable request and with permission of JM.
